# CHRNB4-Mediated Neuroactive Signaling Rewiring Drives Adaptive Resistance to BCL-2 Inhibition in Acute Myeloid Leukemia

**DOI:** 10.3390/cancers18081187

**Published:** 2026-04-08

**Authors:** Hiroaki Koyama, Sachiko Seo, William Tse, Sicheng Bian, Shujun Liu

**Affiliations:** 1 Department of Medicine, The MetroHealth System, Case Western Reserve University, 2500 Metro Health Drive, Cleveland, OH 44109, USA; hxk853@case.edu (H.K.); wwt3@case.edu (W.T.); 2 Department of Hematology, Tokyo Women’s Medical University, 8-1 Kawadacho, Shinjuku, Tokyo 162-8666, Japan; seo.sachiko@twmu.ac.jp; 3 Gene and Cell Therapy Institute (GCTI), The MetroHealth System, Case Western Reserve University, 2500 Metro Health Dr, Cleveland, OH 44109, USA

**Keywords:** acute myeloid leukemia (AML), venetoclax, BCL-2, neuroactive ligand–receptor interaction (NLRI) pathway, CHRNB4, drug resistance

## Abstract

Background: The clinical efficacy of the BCL-2 inhibitor venetoclax in acute myeloid leukemia (AML) is significantly undermined by the frequent emergence of drug resistance, which precipitates disease progression and poor patient outcomes. However, the molecular landscape of this resistance remains insufficiently understood. Methods: To address this, we developed venetoclax-resistant AML cell models and utilized transcriptomic profiling integrated with comprehensive in vitro and in vivo functional assays. Results: Resistant cells demonstrated sustained proliferation even under the suppression of BCL-2, MCL-1, and key intrinsic apoptotic markers, including cleaved PARP and caspase-9, indicating a bypass mechanism independent of classical BCL-2 signaling. Compared to their sensitive counterparts, resistant Kasumi-1 (VENK) and MV4-11 (VENM) cells exhibit aggressive growth phenotypes in vitro and in vivo, characterized by larger, more numerous spheroids and colonies, alongside heightened tumorigenicity in murine models. Transcriptomic profiling and KEGG analysis identified the neuroactive ligand–receptor interaction (NLRI) pathway as a significant signaling node shared between these resistant lines. While multiple NLRI-associated genes were altered, *CHRNB4* was consistently and significantly downregulated in both VENK and VENM cells and tumors. Re-expression of *CHRNB4* in resistant cells, a primary gain-of-function approach, significantly impaired colony formation, and tumor growth in vivo. Clinically, *CHRNB4* downregulation correlates with shortened overall survival and diminished response to venetoclax. Conclusions: Our findings implicate the NLRI pathway in venetoclax resistance and identify CHRNB4 as a robust prognostic indicator and a promising therapeutic target for developing next-generation AML strategies.

## Simple Summary

Despite the success of venetoclax in treating acute myeloid leukemia, the molecular drivers behind emerging drug resistance remain a critical, unsolved hurdle that frequently leads to therapeutic failure. This study uncovers a sophisticated escape mechanism where leukemia cells bypass traditional BCL-2 dependency, adopting a hyperproliferative and aggressive state both in vitro and in vivo. By leveraging transcriptomic profiling, we identified the neuroactive ligand–receptor interaction (NLRI) pathway as a previously unrecognized resistance mechanism. Specifically, the downregulation of *CHRNB4* was pinpointed as a hallmark of resistant cells. Restoring *CHRNB4* expression effectively halted tumor progression, proving its functional significance. These findings carry significant clinical weight, identifying *CHRNB4* as a potent prognostic indicator that predicts patient survival and treatment response, paving the way for innovative, multi-target strategies to overcome resistance in refractory leukemia.

## 1. Introduction

Acute myeloid leukemia (AML) is a complex, diverse disease driven by varied genetic and epigenetic shifts in immature blood cells, resulting in wide-ranging clinical outcomes. While treatment has evolved, the prognosis remains grim for older patients or those harboring high-risk mutations like FLT3-ITD or PTPN11, who often cannot withstand the rigors of intensive chemotherapy [1]. AML survival leans heavily on anti-apoptotic proteins, particularly BCL-2. This vulnerability established BCL-2 inhibitors, notably venetoclax (VEN), as a transformative standard-of-care for patients unfit for traditional chemo, boosting both remission rates and overall survival. Nevertheless, efficacy is not universal; roughly 35% of patients face primary resistance or eventual relapse [2,3]. Mechanisms of resistance are multifaceted and include the co-expression of other anti-apoptotic proteins (e.g., MCL-1, BCL-XL) not targeted by the drug [4,5], the plasticity of the AML cell state leading to adaptive resistance [6,7], and the presence of other high-risk mutations (e.g., FLT3-ITD, PTPN11) [8,9,10]. Combining VEN with other agents (hypomethylating agents (HMAs) like azacitidine or decitabine, or low-dose cytarabine) has improved response rates in older, unfit AML patients [3]. However, overcoming primary resistance and preventing relapse still remain major clinical challenges due to the complex scenarios underlying HMA/VEN treatment [11,12].

The neuroactive ligand–receptor interaction (NLRI) pathway serves as a fundamental signaling axis that modulates critical biological functions. While traditionally associated with neurodegenerative disorders, the NLRI pathway is increasingly recognized for its role in oncogenesis, specifically enhancing tumor aggressiveness and drug resistance in solid malignancies such as prostate and colon cancer [13,14,15,16]. These effects are often mediated through the activation of pro-survival pathways like AKT and NFκB, as well as the modulation of the tumor microenvironment [13,17,18]. Despite these insights in solid tumors, the specific contribution of the NLRI pathway to hematologic malignancies—and particularly the emergence of VEN resistance in AML—remains largely unexplored.

Because NLRI signaling modulates caspase activation—a requisite for VEN-induced apoptosis, we hypothesized that the downregulation of CHRNB4 and the subsequent alteration of NLRI signaling represent a novel survival mechanism that allows AML cells to bypass BCL-2 inhibition. To investigate this, we characterized VEN-resistant AML models across diverse in vitro and in vivo contexts. Through comprehensive molecular and murine analyses, we identified the NLRI pathway as the exclusive shared signaling node significantly dysregulated across independent resistant models. Within this axis, we prioritized CHRNB4 for biological validation, as it represented the sole transcript consistently and significantly suppressed in both cell lines and tumor models. We demonstrate that resistance is mechanistically driven by CHRNB4 silencing. Furthermore, our findings indicate that CHRNB4 depletion serves as a robust predictor of shortened overall survival and diminished clinical response in venetoclax-treated patients. Conversely, enforced CHRNB4 expression—a definitive gain-of-function approach—effectively impairs the clonogenic capacity and tumorigenicity of resistant cells. Collectively, these data establish the NLRI pathway as a mediator of the VEN-resistant phenotype and position CHRNB4 as a critical biomarker for next-generation therapeutic strategies.

## 2. Results

### 2.1. BCL-2 Pathway Alterations Do Not Account for Acquired VEN Resistance

To dissect the molecular mechanisms of acquired VEN resistance, we established drug-resistant derivatives of the AML cell lines MV4-11 and Kasumi-1 via long-term culture with escalating concentrations of VEN (MV4-11: 0.001 to 0.01 nM; Kasumi-1: 0.001 to 0.08 nM). Cells cultured in parallel without drugs served as parental/sensitive controls (hereafter, PAR). We established resistance criteria as routine proliferation in medium containing 0.01 nM (MV4-11) and 0.08 nM (Kasumi-1) VEN, respectively, yielding the resistant cell lines MV4-11-VENR (VENM) and Kasumi-1-VENR (VENK).

**Figure 1:**
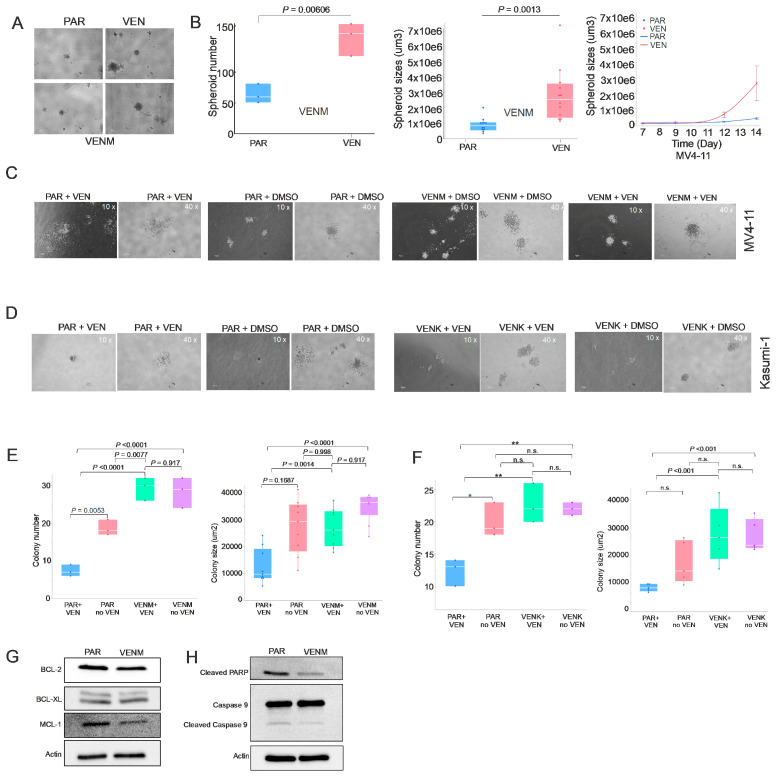
BCL-2 pathway is not required for the survival of VEN-resistant AML cells. (**A**,**B**) Spheroid formation assays of VENM and PAR cells. (**A**) Spheroid forming assays of VENM and PAR cells (day 14). (**B**) Left: Quantification of spheroid number and size on day 14 (mean ± SD). Right: Dynamic changes in spheroid size over the 14-day culture period. (**C**,**D**) Colony formation assays comparing VENM, VENK, and respective PAR cells (day 14), with or without Venetoclax treatment. (**E**,**F**) Quantification of colony number and size on day 14 (mean ± SD). (**G**,**H**) Western blot analysis of caspases and anti-apoptotic proteins in VENM cells compared to PAR cells. Results represent three independent experiments. Note: VEN, venetoclax; VENM, MV4-11 VEN-resistant cells; VENK, Kasumi-1 VEN-resistant cells; PAR, parental/sensitive cells; n.s., not statistically significant; * *p* < 0.05; ** *p* < 0.01.

First, we evaluated the cells’ capacity to form spheroids—three-dimensional structures that better mimic bone marrow microenvironment (BME) interactions [19]. While parental (PAR) MV4-11 cells produced irregular, dendritic-like clusters with weak cohesion, the resistant cells generated significantly more and larger spheroids (Figure 1A,B). These resistant clones also displayed tighter cell–cell adhesion and maintained a dense, spherical shape regardless of VEN exposure. Interestingly, we observed no spheroid formation in either the PAR or VENK groups. Next, we tested colony-forming potential. Under VEN treatment, PAR cells showed minimal colony growth and poor adhesion. Conversely, both VENM and VENK variants produced more abundant, larger colonies with clearly distinct morphologies (Figure 1C,D). Resistant clones maintained this robust colony-forming potential even after VEN treatment, showing no significant changes in colony morphology or number, which confirms their resistance to VEN. Third, we confirmed the acquisition of resistance via drug sensitivity assays. While VEN treatment significantly reduced, or showed a trend toward reducing, the colony number and size of PAR cells, both the VENM and VENK cells showed minimal changes in colony number and size, indicating reduced sensitivity (Figure 1E,F). These results suggest that selective pressure from VEN enhances the oncogenic potential and drug resistance of AML cells.

To investigate the mechanisms underlying these VEN-altered phenotypes, we performed Western blot analysis in VENM cells. We found that the expression levels of the anti-apoptotic proteins BCL-2 and MCL-1 are decreased compared to PAR cells; notably, BCL-xL levels remained unchanged (Figure 1G and Figure S1A,B). Furthermore, the levels of cleaved PARP and caspase-9 were also decreased, indicating an inhibition of the caspase pathway in the VEN-resistant cells (Figure 1H and Figure S1C,D). These results suggest that the acquisition of VEN resistance is accompanied by a shift toward alternative survival pathways rather than continued dependence on the canonical BCL-2 pathway.

### 2.2. Resistant VENM Cells Demonstrate Aggressive Growth Advantages In Vivo

**Figure 2:**
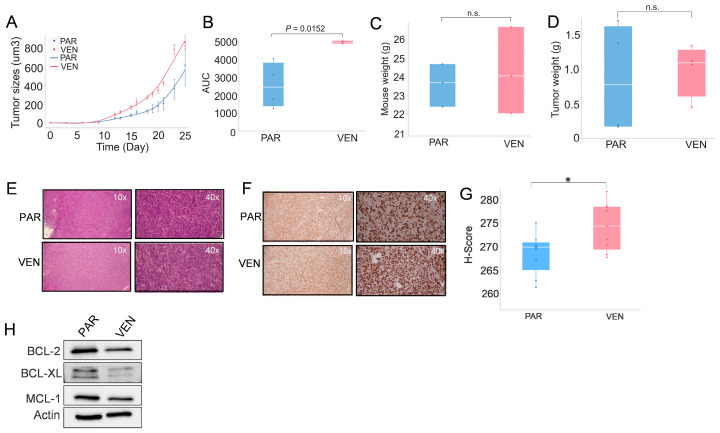
VEN-resistant AML cells exhibit increased tumorigenicity in vivo. PAR and VENM cells were subcutaneously injected into the flanks of nude mice to assess tumor growth and properties (*n* = 4 tumors/group). (**A**) Tumor volume over a 25-day period, showing dynamic changes in size. (**B**) Quantification of tumor growth represented as Area Under the Curve (AUC) values over the 25 days. (**C**,**D**) Host body weight (**C**) and excised tumor weight (**D**) at the study endpoint (day 25). (**E**,**F**) Representative images of tumor sections analyzed by H&E staining (**E**) or IHC using an anti-Ki-67 antibody (**F**). All images were captured at ×200 magnification (*n* = 3). (**G**) Quantitative analysis of the Ki-67 IHC results. (**H**) Western blot analysis of BCL-2, BCL-XL, and MCL-1 within the harvested tumor tissues. VEN, venetoclax; VENM, MV4-11 VEN-resistant cells; H&E, hematoxylin and eosin; IHC, immunohistochemistry staining; PAR, parental/sensitive cells. n.s., not significant; * *p* < 0.05.

To evaluate whether VENM cells exhibit enhanced fitness in vivo, 3 × 10^6^ cells from each group were injected subcutaneously into the flanks of nude mice following a 72 h recovery period in drug-free medium. Both cohorts displayed equivalent engraftment rates (67%). Notably, VENM-derived tumors exhibited an accelerated growth trajectory during the initial phase compared to PAR-derived tumors. While the proportional rate of volume increase eventually stabilized between groups after day 8, this early kinetic surge resulted in a consistently higher cumulative tumor burden in the VENM cohort throughout the 25-day study (Figure 2A).

To precisely capture these growth dynamics, we utilized the Area Under the Curve (AUC) as a robust indicator of tumor ‘tempo’ and cumulative progression. The mean AUC was significantly elevated in the VENM group (4932.9 ± 81.5) compared to the PAR group (2540.0 ± 1276.0; *p* = 0.033) (Figure 2B). While the final mean tumor volume (860.1 ± 73.6 mm^3^ vs. 615.4 ± 218.3 mm^3^; *p* = 0.35) and terminal tumor weight (976 ± 188 mg vs. 840 ± 395 mg; *p* = 0.77) followed a similar upward trend, the statistical significance of the AUC underscores a more aggressive overall growth profile in VENM-derived tumors. No significant variations in mouse body weight were observed (Figure 2C). This increased in vivo aggressiveness aligns with the enhanced proliferative capacity observed in vitro, characterized by larger spheroid and colony formation. The hyper-proliferative phenotype of VENM tumors was further confirmed by more robust H&E staining (Figure 2E) and significantly higher expression of the proliferation marker Ki-67 (Figure 2F,G) when compared with the PAR tumors.

To investigate the mechanisms underlying this enhanced tumorigenesis, we performed Western blotting. The results revealed that the expression of the anti-apoptotic markers BCL-2, MCL-1, and BCL-xl is slightly decreased in VENM vs. control tumors (Figure 2H and Figure S2A,B), consistent with our in vitro findings. Taken together, these data indicate that VENM cells acquire more aggressive phenotypes in vivo, independent of BCL-2 pathway.

### 2.3. Chronic VEN Exposure Drives a Comprehensive Rewiring of the AML Transcriptome

**Figure 3:**
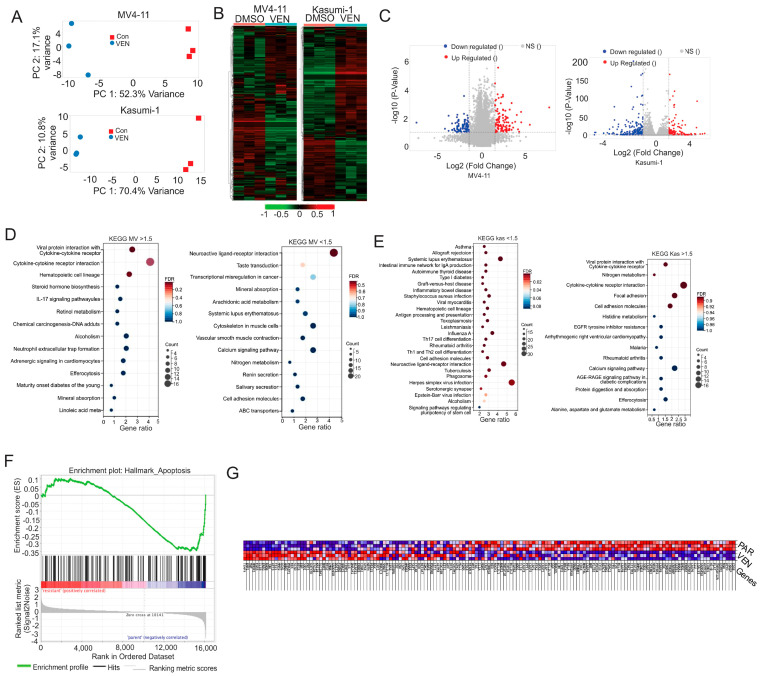
Selective pressures imposed by VEN significantly alter the transcriptomic landscapes in AML cells. (**A**) PCA of the global gene expression profiles of all analyzed samples, demonstrating the distinct separation between PAR and VENM groups along PC1 and PC2. (**B**) Heatmaps of DETs between treatment groups. (**C**) Volcano plot visualizing DETs based on statistical significance and magnitude of change. Transcripts significantly upregulated (red) or downregulated (blue) in VENM cells compared to PAR are highlighted. The *x*-axis represents the log_2_ fold change (log_2_FC), and the *y*-axis represents the negative log_10_ adjusted *p*-value (−log_10_ padj); dashed lines indicate significance thresholds (padj < 0.05 and |log_2_FC| > 1). (**D**,**E**) KEGG pathway enrichment analysis. Bubbles display the top enriched pathways ranked by significance (*p*-value). Note: These panels utilize independent axis scales to ensure the visibility of enrichment trends within each condition; therefore, they are not intended for direct quantitative comparison. (**F**) GSEA plot of enriched hallmark pathways and their leading-edge gene subsets. (**G**) Heatmap of leading-edge gene expression, with values ranging from red (highest) to dark blue (lowest). PCA, Principal Component Analysis; DETs, differentially expressed transcripts; GSEA, Gene Set Enrichment Analysis; FC, fold change; FDR, False Discovery Rate.

To further elucidate the molecular rules of VEN resistance, we performed bulk RNA sequencing on VEN-resistant cells (VENM, VENK) and their respective PAR cells. Transcriptome-wide analyses demonstrated high concordance among triplicates and a clear separation of gene expression profiles between PAR and resistant cell populations (Figure 3A). Unsupervised hierarchical clustering of the data revealed 2000 significantly differentially expressed transcripts (DETs; false discovery rate (FDR); adjusted *p* ≤ 0.05) across the groups (Figure 3B; Supplementary Tables S1 and S2). VEN exposure led to substantial transcriptomic changes in both cell lines: in VENM cells, VEN resistance resulted in the upregulation of 1075 transcripts (6.7% of total detected transcripts; fold change [FC] range: 0.00014 to 7.78683) and the downregulation of 2342 transcripts (14.6%; FC range: 0.00042 to 7.59844). In VENK cells, we observed the upregulation of 2219 transcripts (14.2%; FC range: 0.083866 to 5.670289) and the downregulation of 2509 transcripts (16.1%; FC range: 0.08855 to 7.11745) (Figure 3C). The top 100 most significantly downregulated and upregulated transcripts (Q < 0.05) are presented in Supplementary Tables S3 and S4.

Functional consequences of the observed transcriptional changes were investigated using KEGG pathway enrichment analysis (DAVID bioinformatics resource) and gene set enrichment analysis (GSEA). The KEGG analysis revealed significant enrichment in several cancer-associated pathways, including NLRI pathway (Figure 3D,E; Supplementary Table S5). The GSEA, specifically the hallmark analysis, showed that VEN exposure results in 15 activated and 35 inhibited gene sets. Notably, the hallmark apoptosis gene set was suppressed while the PI3K-AKT-mTOR pathway was activated (Figure 3F,G). These results collectively indicate that acquired VEN resistance in AML cells involves substantial transcriptomic remodeling, promoting cell survival via the suppression of apoptosis and the activation of oncogenic pathways identified across both DAVID-KEGG and GSEA platforms.

### 2.4. Systematic Profiling Identifies Dysregulation of the NLRI Pathway

**Figure 4:**
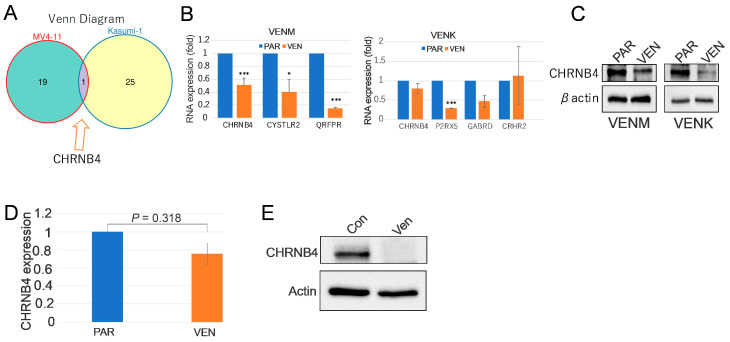
Reduced CHRNB4 expression in in vitro and in vivo VENM models. (**A**) Venn diagram of the overlap of DEGs between VENM and VENK cells. (**B**,**C**) qPCR (**B**) and Western blot (**C**) analysis demonstrating the relative expression levels of the indicated genes (specifically including CHRNB4) in resistant cell lines. The Western blot results represent the findings from three independent experiments. (**D**,**E**) qPCR (**D**) and Western blot (**E**) of indicated genes in tumors. The results in Western blot represent three independent experiments. VEN, VEN-resistant cells; VENM, VEN-resistant MV4-11 cells; VENK, VEN-resistant Kasumi-1 cells. * *p* < 0.05; *** *p* < 0.001.

Transcriptomic analysis revealed the NLRI pathway as the most significantly dysregulated among the enriched sets in both VENM and VENK cells. This pathway involved 20 genes in the VENM group and 26 in the VENK group (Supplementary Table S6), with CHRNB4 emerging as a central component shared by both (Figure 4A). We performed qPCR to validate expression shifts in specific NLRI-associated genes. In VENM cells, we analyzed CHRNB4, CYSLTR2, and QRFPR; in VENK cells, we tracked CHRNB4, P2RX5, GABRD, and CRHR2. Our results showed significant downregulation of CHRNB4, CYSLTR2, and QRFPR in VENM cells compared to the PAR control. Conversely, while P2RX5 was significantly lower in VENK cells, CHRNB4 and GABRD only showed a downward trend, and CRHR2 remained unchanged (Figure 4B). To confirm these findings at the protein level, we focused on the key marker CHRNB4. Western blotting revealed a marked reduction in CHRNB4 protein across both resistant lines (Figure 4C and Figure S3A,B). Crucially, this decline in both RNA and protein was also observed in VENM-derived tumors (Figure 4D,E and Figure S3C,D). Taken together, these data suggest that NLRI pathway dysregulation—specifically the suppression of CHRNB4—may drive VEN resistance.

### 2.5. Loss of CHRNB4 Expression Emerges as a Key Regulator of VEN-Resistant Phenotype

**Figure 5:**
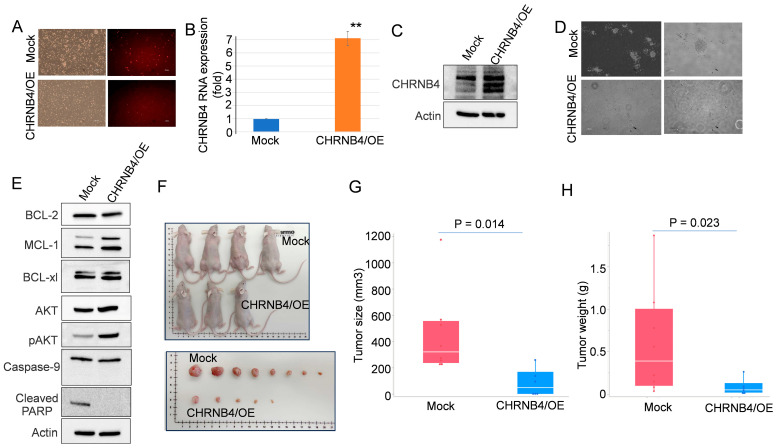
Functional and transcriptomic consequences of CHRNB4 restoration in VEN-resistant AML. (**A**) Successful CHRNB4 overexpression was confirmed in VENM cells following lentiviral infection with a CHRNB4 expression vector and subsequent selection using 2 µg/mL puromycin. Representative fluorescence microscopy images of the linked reporter. Scale bar: 100 µm. (**B**,**C**) qPCR (**B**) and Western blot (**C**) analysis confirming CHRNB4 mRNA and protein upregulation in the cells described in (**A**). (**D**) Colony-formation assays were performed to assess the impact of sustained CHRNB4 expression on cell proliferation and clonogenic survival, comparing CHRNB4-overexpressing cells to control cells. Scale bar: 100 µm. (**E**) Western blot analysis of CHRNB4 and relevant indicated target genes in overexpressing cells relative to controls. The immunoblots shown are representative of results obtained from three independent experiments. (**F**) Macroscopic images of tumor-bearing mice and dissected tumors (*n* = 8 per group) at the study endpoint. (**G**,**H**) Quantification of tumor weight (**G**) and tumor volume trajectories (**H**) over time. ** *p* < 0.01.

We focused on the NLRI pathway and the gene CHRNB4 to define their role in venetoclax resistance. This choice was driven by two key factors: first, CHRNB4 was the only common gene altered in both VENM and VENK lines (see Figure 4A); second, while its role in smoking-related cancers like HNSCC and ESCC is documented [20,21,22], its contribution to leukemia drug resistance remained unknown. To test the functional impact of CHRNB4, we overexpressed it in VENM cells, which normally show low endogenous levels (Figure 5A). We confirmed successful expression via qPCR and Western blot (Figure 5B,C and Figure S4A,B). Colony-forming assays then revealed that restoring CHRNB4 expressions resulted in a distinct morphological change, specifically, larger clones with “sprayed over” or more dispersed cell aggregates and colony-forming ability compared to controls (Figure 5D). Notably, this inhibitory effect persisted regardless of VEN treatment, suggesting that restored CHRNB4 function itself drives the observed suppression.

Previous work suggests CHRNB4 regulates survival through PI3K/Akt/mTOR signaling or apoptosis inhibition [20,21]. To probe this in our model, we performed Western blots on the overexpressing cells. Surprisingly, this showed increased pAKT and MCL1 levels alongside decreased cleaved PARP, with no change in cleaved caspase-9 (Figure 5E and Figure S4C,D). While these markers typically signal robust survival and suppressed apoptosis, our functional assays presented a clear paradox: despite these pro-survival biochemical shifts, colony formation and adhesion were severely impaired. This indicates a significant dissociation between classical survival signaling and the actual cellular dysfunction triggered by CHRNB4.

### 2.6. Restoring CHRNB4 Expression Suppresses Tumor Growth in VEN-Resistant AML Models

To evaluate the functional role of CHRNB4 in vivo, we stably overexpressed CHRNB4 in VEN-resistant MV4-11 cells, which are characterized by low endogenous CHRNB4 levels. We subcutaneously inoculated 3 × 10^6^ cells into the bilateral flanks of 6-week-old athymic nude mice (*n* = 4 per group). Restoring CHRNB4 markedly reduced tumor incidence to 62.5% (5/8 sites), compared to 100% (8/8 sites) in the mock-transduced cohort (Figure 5F). One animal in the overexpression group was humanely euthanized before the endpoint due to a non-study-related skin wound and was excluded from the final analysis. Beyond reducing incidence, CHRNB4 overexpression significantly suppressed the growth of established tumors. Post-resection analysis revealed substantial reductions in both tumor volume (451.68 ± 113.50 mm^3^ mock vs. 81.56 ± 42.61 mm^3^ OE) and tumor weight (0.59 ± 0.22 g mock vs. 0.078 ± 0.039 g OE) (Figure 5G,H). These data suggest that CHRNB4 serves as a potent tumor suppressor in the context of acquired VEN resistance.

### 2.7. Low CHRNB4 Levels Correlate with Poor Clinical Outcomes and Reduced VEN Response in Leukemia Patients

**Figure 6:**
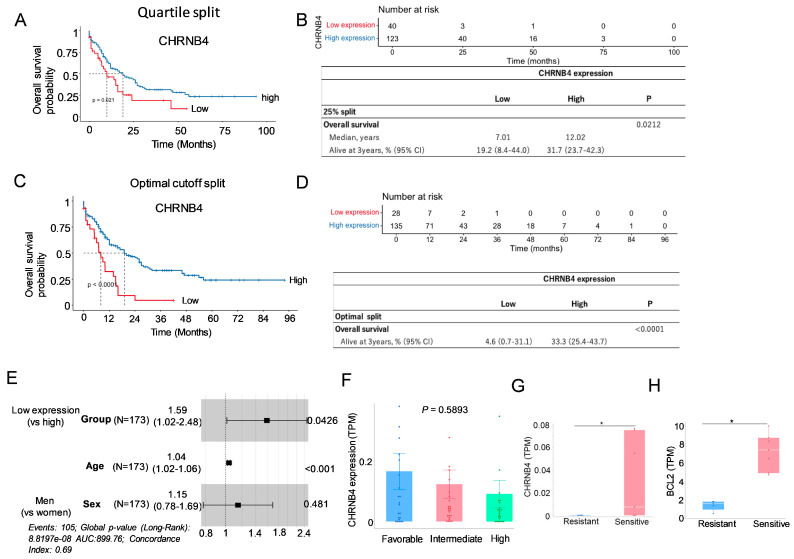
CHRNB4 expression predicts AML prognosis and correlates with VEN response. (**A**–**D**) Analysis of AML patient data from the TCGA Firehose Legacy study (*n* = sample size). (**A**) Kaplan–Meier (KM) survival curve demonstrating significantly poorer overall survival in patients with low CHRNB4 expression (≤25th percentile). (**B**) Forest plot identifying low CHRNB4 expression as an independent adverse prognostic factor. (**C**,**D**) KM curves confirming consistent OS trends when data is stratified by median split (**C**) and optimal cut-off (**D**). (**E**) Hazard ratios and 95% confidence intervals for overall survival from patient group with low expression of CHRNB4 compared to those with high CHRNB4 expression. (**F**) Comparison of CHRNB4 expression across favorable, intermediate, and poor prognostic subgroups (defined by FLT3/NPM1 status). (**G**,**H**) Analysis of RNA-sequencing data from an independent cohort of 12 AML clinical samples (GSE132511). (**G**) Correlation between CHRNB4 and BCL-2 expression levels. (**H**) Association between CHRNB4 expression and clinical response to VEN treatment. * *p* < 0.05.

To assess the clinical significance of CHRNB4 in AML, we analyzed the TCGA Firehose Legacy AML cohort (*n* = 173). Patient characteristics are presented in Supplementary Table S7. Kaplan–Meier survival analysis revealed a significant association between low CHRNB4 expression levels and shorter overall survival (OS) (*p* = 0.021) (Figure 6A–E). Specifically, patients in the lowest quartile (25%) for CHRNB4 expression had a median OS of 7.01 months, compared with 12.02 months for patients with higher expression levels. Furthermore, a multivariate Cox proportional hazards regression confirmed that low CHRNB4 expression is an independent adverse prognostic factor in AML (Hazard Ratio [HR] = 1.59, 95% Confidence Interval [CI] = 1.0154–2.476, *p* = 0.0426). To verify the consistency of these findings, we performed additional survival analyses using both median and optimal cut-offs (determined via maximally selected rank statistics), all of which consistently demonstrated poorer survival in the low-expression group. These results are further supported by the observation that CHRNB4 expression is downregulated in high-risk AML patients (e.g., those with FLT3-ITD without NPM1 co-mutation) compared with favorable-risk patients (NPM1 without FLT3-ITD co-mutation) (Figure 6F). Together, these findings suggest that the downregulation of CHRNB4 may play a critical role in mediating VEN resistance in AML.

The role of CHRNB4 in regulating sensitivity to VEN was investigated using RNA-seq data (GSE132511) from 12 AML clinical samples. Samples were categorized into VEN-sensitive (monocytic lineage, *n* = 5) and VEN-resistant (primitive lineage, *n* = 7) groups based on the established criteria [11]. Comparative analysis revealed that patients with higher BCL2 expression are more sensitive to VEN therapy compared to those in the lower-expression group (Figure 6G). Furthermore, CHRNB4 expression was significantly lower in the VEN-resistant group compared to the sensitive group (*p* = 0.0332; Figure 6H). This differential expression suggests that the downregulation of CHRNB4 may contribute to the mechanism of VEN resistance, highlighting its potential impact on drug efficacy in vivo.

## 3. Discussion

While venetoclax (VEN) monotherapy initially showed promise, its clinical utility in AML is severely limited by the inevitable emergence of acquired resistance [23,24]. Although FDA-approved combinations with hypomethylating agents or low-dose cytarabine have improved remission rates and overall survival [23,25,26,27,28], significant hurdles remain, including profound myelosuppression and persistent therapeutic evasion [26,29]. This study elucidates a novel molecular driver of such resistance, demonstrating that chronic VEN exposure triggers a transcriptomic rewiring of the NLRI pathway. Specifically, we identified the downregulation of CHRNB4 as a critical mediator of reduced drug sensitivity. These findings position CHRNB4 as a potential indicator for VEN response and suggest that targeting the NLRI axis offers a compelling therapeutic strategy to overcome resistance in AML.

Resistance to VEN in leukemia is inherently multifactorial, typically driven by molecular adaptations that reinforce anti-apoptotic defenses. Standard mechanisms include the upregulation of survival proteins like MCL-1 and BCL-XL, acquired mutations in BCL2 or BAX, and the activation of compensatory signaling through the PI3K/AKT axis [30]. We prioritized the MV4-11 (VENM) model for in-depth mechanistic and in vivo studies because its FLT3-ITD mutation more accurately reflects the high-risk genetic landscape seen in clinical VEN resistance. Additionally, this model’s reliable engraftment and well-characterized growth kinetics provided a robust, reproducible system for our xenograft and molecular analyses. Consistent with previous reports [31,32], our data demonstrate that resistant VENM and VENK cells possess superior proliferative fitness, characterized by increased colony density and spheroid size. Furthermore, in vivo xenograft models confirmed that VENM cells harbor significantly higher tumorigenic potential than their parental counterparts. However, our mechanistic findings deviate from the classical paradigm. Rather than an increase, we observed a subtle decrease in MCL-1 and BCL-XL expression in resistant cells—a shift that, in isolation, should lower the apoptotic threshold. Paradoxically, this was accompanied by a marked reduction in cleaved caspase-9 and PARP, indicating a profound inhibition of the executioner caspase cascade. Collectively, these results suggest that VEN resistance in this context involves a compensatory shift toward BCL-2-independent survival mechanisms. This discovery provides a strong rationale for combining VEN with agents like HMAs, which can further suppress MCL-1 and BCL-XL, induce pro-apoptotic NOXA, and reverse the epigenetic silencing of genes such as GSDME to bypass intrinsic resistance.

Mutations in FLT3 and KIT are established drivers of VEN resistance in AML, frequently correlating with diminished therapeutic response and a higher propensity for clinical relapse [8,9,10,33,34]. Our analysis reveals that CHRNB4 expression is significantly downregulated in high-risk patient cohorts—such as those harboring FLT3-ITD without NPM1 mutations—compared to those with favorable risk profiles. These data suggest a potential synergy between CHRNB4 depletion and oncogenic FLT3 or KIT signaling in the establishment of resistant leukemic populations. Notably, while the specific NLRI pathway components dysregulated in our models varied (20 genes in VENM vs. 26 in VENK), CHRNB4 emerged as the exclusive overlapping node across these genetically distinct backgrounds. This ‘pathway-level convergence’—where different genetic nodes within the same network are recruited depending on the oncogenic context—is a recognized phenomenon in cancer systems biology. The consistent suppression of CHRNB4 across both FLT3-mutated and KIT-mutated lineages suggests it may represent a critical common denominator, or ‘master regulator,’ of the resistant phenotype. Furthermore, the non-overlapping genes identified likely reflect the biological plasticity and functional redundancy inherent in AML, where complementary signals within the NLRI pathway bypass BCL-2 inhibition. While this study prioritizes the functional validation of CHRNB4, the broader transcriptomic remodeling observed underscores the complexity of this bypass mechanism. Future investigations into these secondary NLRI-associated genes will further elucidate the molecular framework of resistance and may uncover therapeutic vulnerabilities tailored to the specific genetic context of FLT3- or KIT-mutant AML.

The NLRI pathway is a critical signaling scaffold that, while primarily known for regulating neurobiological functions, is increasingly implicated in tumor progression and therapeutic evasion across diverse malignancies [13,14,15,16]. Although mechanisms such as the activation of pro-survival signaling, neuroendocrine differentiation, and epigenetic remodeling have been documented in solid tumors, a significant knowledge gap persists regarding how this pathway drives resistance in hematological malignancies, particularly against VEN in AML. To address this, we performed transcriptomic profiling of VEN-resistant (VENM and VENK) and PAR cells. Integrated KEGG and GSEA analyses identified the NLRI and apoptotic pathways as the most significantly enriched among differentially expressed transcripts. Specifically, the NLRI pathway involved 20 altered genes in VENM and 26 in VENK cells. Notably, CHRNB4 emerged as the only shared gene consistently downregulated across both resistant models and their derived tumors, suggesting a central regulatory role in VEN resistance. These resistant populations exhibited a markedly aggressive phenotype, characterized by accelerated in vitro proliferation, enhanced spheroid and colony-forming capacity, and heightened tumorigenicity in vivo. Crucially, the functional restoration of CHRNB4 in resistant cells significantly impaired both cell adhesion and colony formation. Clinical validation further underscored these findings, as lower CHRNB4 expression in AML patients predicted significantly shorter overall survival and a diminished response to VEN-based regimens. Collectively, our data establish the NLRI pathway as a primary driver of VEN resistance and identify CHRNB4 as both a potential prognostic indicator and a promising therapeutic target for overcoming resistance in AML.

Analysis of TCGA data reveals that low CHRNB4 expression correlates with poor clinical prognosis and molecular signatures of venetoclax resistance, including elevated pAKT and reduced BCL-2/MCL-1 ratios. These findings suggest that CHRNB4 downregulation facilitates both disease progression and pro-survival signaling. Conversely, our functional assays show that CHRNB4 overexpression impairs tumor formation and suppresses growth, reinforcing its role as a putative tumor suppressor.

Our overexpression experiments revealed a notable discrepancy: while CHRNB4 restoration was associated with increased survival markers like pAKT and MCL-1, it simultaneously inhibited colony formation and adhesion without inducing PARP cleavage. This suggests that despite the induction of certain survival signals, the overall fitness of these leukemic cells is significantly compromised. A possible explanation for this “signaling-to-function” divergence is that high-level CHRNB4 expression may correlate with cellular stress, such as endoplasmic reticulum (ER) stress or altered Ca^2+^ homeostasis [35,36]. These transcriptomic signatures might reflect a state where compensatory upregulation of pAKT and MCL-1 occurs alongside a reduction in proliferative and adhesive capacity [37,38,39]. In this context, the absence of cleaved PARP suggests that the cells may be undergoing functional impairment rather than immediate apoptosis. Further investigation is required to definitively characterize these stress-mediated pathways and their role in modulating the fitness of venetoclax-resistant AML.

Our study has several limitations. First, while our results highlight the impact of CHRNB4 on growth dynamics, the study is restricted to two AML cell lines. Given the limited gene-level overlap between these models, the claim of a shared NLRI-driven resistance program across diverse AML contexts must be interpreted with caution. Furthermore, “resistance” in our models was primarily defined by sustained proliferation under continuous selective pressure rather than formal IC50 or AUC shifts. While our chosen drug concentrations (0.01–0.08 nM) align with the estimated physiologically active, unbound fraction of venetoclax (~0.2 nM) [40], future studies incorporating standard dose–response analyses and broader genomic representation are required to ensure these mechanisms are universally applicable across the heterogeneous landscape of AML.

Second, our mechanistic evidence is currently supported primarily in a single model and based on gain-of-function re-expression. The lack of complementary loss-of-function data (knockdown/knockout) in parental cells, alongside the absence of direct chemosensitivity rescue and apoptosis assays, remains a significant limitation. While CHRNB4 suppresses aggressive growth phenotypes, we have not yet definitively quantified its direct impact on the apoptotic threshold or BCL-2 dependency through biophysical assays like BH3 profiling. Moreover, the observed signaling-to-function discrepancy—where survival markers increased despite inhibited growth—suggests complex, stress-mediated contradictions that remain to be fully resolved.

Third, our in vivo and 3D Matrigel models did not evaluate tumor growth under continuous drug exposure. Because these assays were conducted without immediate post-injection drug exposure, they should be framed as exploratory rather than definitive evidence of therapeutic resistance. Investigating these kinetics with active drug pressure will be critical to precisely characterize the fitness and evolutionary trajectory of resistant clones. Additionally, as no formal blinding or randomization was applied to these procedures, a potential risk of observer bias must be acknowledged.

Finally, our patient-level validation is preliminary and utilized a cohort where CHRNB4 expression may partially reflect underlying differentiation states, such as primitive versus monocytic maturation. Larger, prospective clinical cohorts are essential to confirm the independent prognostic value of CHRNB4 and establish its utility as a robust biomarker. Collectively, these points identify the necessary trajectory for future research to refine the role of the NLRI pathway in VEN-resistant AML.

## 4. Experimental Methods

### 4.1. Cell Lines and Cell Culture

The human AML cell lines MV4-11 (CRL-9591) and Kasumi-1 (CRL-2724) were obtained from the American Type Culture Collection (ATCC). MV4-11 is a non-adherent line carrying the t(4;11) translocation (MLL-AF4 fusion) and a FLT3-ITD mutation. Kasumi-1, derived from an AML patient with the t(8;21) translocation (RUNX1-RUNX1T1/AML1-ETO fusion), harbors a c-KIT-activating mutation and exhibits myeloid and macrophage lineage characteristics. While we relied on the characterization and quality control provided by the vendor at the time of purchase, we acknowledge that additional in-house authentication (STR profiling) or mycoplasma testing was not performed for this study. To mitigate risks of contamination or misidentification, both lines were verified against the International Cell Line Authentication Committee (ICLAC) database and were not found among the registers of commonly misidentified or cross-contaminated cell lines.

Cells were cultured in RPMI-1640 medium (GE Healthcare #SH30027.01, GE Healthcare, Uppsala, Sweden) supplemented with 10% (for MV4-11) or 20% (for Kasumi-1) fetal bovine serum (FBS, Gibco by Life Technologies #16140-071, Thermo Fisher Scientific, Waltham, MA, USA) and 1% Antibiotic–Antimycotic (Gibco by Life Technologies #15240062, Thermo Fisher Scientific, Waltham, MA, USA). Cells were maintained at 37 °C in a humidified incubator with 5% CO_2_.

### 4.2. Reagents and Plasmid Constructs

The BCL-2 inhibitor Venetoclax (HY-15531) was purchased from MedChemExpress (Princeton, NJ, USA). For in vitro studies, all drugs were initially dissolved in dimethyl sulfoxide (DMSO) and stored at −80 °C. The CHRNB4 overexpression plasmid was obtained from Vector Builder (Chicago, IL, USA), and the corresponding control plasmid was acquired from Addgene (Watertown, MA, USA).

### 4.3. Stepwise Induction of Venetoclax Resistance

Parental MV4-11 and Kasumi-1 cells were exposed to a low, sub-lethal concentration of the BCL-2 inhibitor VEN (0.001 nM) in a stepwise manner to induce resistance. Surviving cells were cultured, and upon recovery of consistent growth rates, the venetoclax concentration was incrementally increased. Final concentrations reached 0.01 nM for MV4-11 and 0.08 nM for Kasumi-1 cells. The total selection period was 8–10 weeks. Cell viability, proliferation rate, and morphological changes were routinely monitored during the adaptation process. The ‘resistance’ in this study is defined as the sustained proliferative capacity of VENK and VENM cells under continuous VEN exposure, which resulted in the inhibition of the parental lines. Once established, resistant cell lines were routinely maintained in medium containing 0.01 nM (MV4-11) and 0.08 nM (Kasumi-1) venetoclax to preserve the resistant phenotype. For experiments requiring drug-free conditions, both parental and resistant cells were washed once with blank RPMI 1640 medium (no FBS, no antibiotics) to remove any residual venetoclax. The washed cells were then cultured in regular RPMI 1640 medium (with 10% FBS and antibiotics) for 48–72 h to allow for recovery and proliferation in a drug-free environment before use in downstream assays. The 72 h recovery period was intentionally designed to ensure that both parental and resistant cells were in a comparable physiological state at the time of injection. This brief window allows for the clearance of residual VEN, ensuring that any observed differences in growth are due to the stable, acquired phenotypes of the resistant cells rather than acute, transient drug effects.

### 4.4. Lentivirus Vector, Virus Production and Virus Infection

For lentivirus production, HEK-293T cells were seeded in a 10 cm culture dish at a density of 2.0 × 10^6^ cells/dish and incubated for 24 h. Cells were subsequently transfected with 4 µg of either the target or scrambled control plasmids using a CalPhos™ Mammalian Transfection Kit (Clontech Laboratories, Inc., Mountain View, CA, USA), following the manufacturer’s protocol. Viral supernatants were collected at both 48 and 72 h post-transfection and concentrated using a Lenti-X™ Concentrator (Clontech, Cat. #631232). For infection, 2 × 10^6^ recipient MV4-11-resistant cells were resuspended in 1 mL of medium and transduced with the harvested lentiviruses in the presence of Polybrene at a final concentration of 4 µg/mL. Stable transformants were selected by adding puromycin to a final concentration of 1 µg/mL 24 h post-transduction.

### 4.5. Colony-Forming Assays

Progenitor cell function was assessed via colony-forming assays using Human Methylcellulose Complete Media (R&D Systems, #HSC003, Bio-Techne Corporation, Minneapolis, MN, USA). Briefly, cells were suspended in 0.3 mL of the provided cell suspension medium and then thoroughly mixed with 3 mL of the complete methylcellulose media. This mixture was plated into 35 mm dishes and incubated. Colony number and size were evaluated and recorded after 7–14 days.

### 4.6. 3D Spheroid-Forming Assays

For spheroid assays, 2000 cells were suspended in 100 µL of Corning Matrigel Matrix Growth Factor Reduced (Corning #356230, Corning Life Sciences, Tewksbury, MA, USA) and seeded on the 24-well culture plate. The spheroids were then cultured for 3 to 14 days under standard conditions. Imaging was performed using a Zeiss LSM 880 confocal/multiphoton microscope (Carl Zeiss AG, Oberkochen, Germany). Two-photon excitation was achieved with an 800 nm laser, and fluorophores were spectrally separated by their emission for detection. Note that spheroid assays are intended to model the protective effects of the bone marrow niche rather than traditional epithelial spheroids.

### 4.7. Western Blotting

The whole cellular lysates were prepared by harvesting the cells in 1 × cell lysis buffer [20 mM HEPES (pH 7.0), 150 mM NaCl and 0.1% NP40] supplemented with 1 mM phenylmethane sulfonyl fluoride (PMSF, Sigma #10837091001, St. Louis, MO, USA), 1 × Phosphatase Inhibitor Cocktail 2 and 3 (Sigma #P5726, P0044), and 1 × protease inhibitors (protease inhibitor cocktail set III, Calbiochem–Novabiochem #539134, San Diego, CA, USA). The proteins were resolved by sodium dodecyl sulfate (SDS)–polyacrylamide gel electrophoresis, transferred onto PVDF membranes (GE Healthcare #10600023), blocked by 5% non-fat milk followed by probing with first antibodies, anti-BCL-2 (Millipore, St. Louis, MO, USA, #05-729), anti-BCL-XL (Santa Cruz, Dallas, TX, USA, #sc-7195), anti-MCL-1 (Santa Cruz, #sc-819), anti-AKT (Cell Signaling, Danvers, MA, USA, #9272), Anti-pAKT (Cell Signaling, #4060), anti-CHRNB4 (Abcam, Cambridge, UK, #ab233735), anti-β-Actin (Cell Signaling, #4967) as well as HRP-conjugated secondary antibodies, goat anti-rabbit IgG (Cell Signaling, #7074), and goat anti-mouse IgG (Cell Signaling, #7076).

### 4.8. RNA Isolation, cDNA Preparation and Quantitative PCR (qPCR)

Total RNA was isolated according to the manufacturer’s instructions using the miRNeasy Kit (Qiagen, Hilden, Germany, #217004). Complementary DNA (cDNA) synthesis was synthesized from the isolated RNA using SuperScript^®^ III First-Strand Synthesis System (Invitrogen, Carlsbad, CA, USA, #18080-051). Gene expression levels were quantified via qPCR using the SYBR Green Master Mix (Applied Biosystems, Foster City, CA, USA, #4309155). Target gene expression was normalized to 18S ribosomal RNA levels. Primer sequences are CHRNB4: F 5′-CAGCTTATCAGCGTGAATGAGC-3′, R 5′-GTCAGGCGGTAATCAGTCCAT-3′; CYSLTR2: F 5′-ACTGAGGACCGTCCACTTGA-3′, R 5′-CCCAGCAAAGTAATAGAGCAGAG-3′; QRFPR: F 5′-CAGGCGCTTAACATTACCCC-3′, R 5′-CCGGTACAGAGCGATGAACTG-3′; P2RX5: F 5′-TACCTGGTCGTATGGGTGTTC-3′, R 5′-GCCCAAGATCCGAGGTGTTG-3′; GABRD: F 5′-GCATCCGAATCACCTCCACTG-3′, R 5′-GATGAGTAACCGTAGCTCTCCA-3′; CRHR2: F 5′-CCCTTGTCGTCAACTACCTGG-3′, R 5′-ACATTTCGCAGGATAAAGGTGG-3′; 18S: F 5′-ATTAAGGGTGTGGGCCGAAG-3′, R 5′-TGGCTAGGACGTGGCTGTAT-3′.

### 4.9. RNA Sequencing and Data Processing

Strand-specific transcriptome library construction was completed by enriching mRNA from total RNA, and sequenced by the DNBSEQ high-throughput platform at BGI (https://www.bgi.com). Briefly, mRNA was purified from total RNA using oligo(dT) magnetic beads, fragmented, and converted to double-stranded cDNA with dUTP incorporated into the second strand. The cDNA underwent end repair, A-tailing, and adapter ligation. UDG digestion was performed to remove the second strand before PCR amplification. After purification with XP Beads (Beckman Coulter Life Sciences, Indianapolis, IN, USA), the library was validated on an Agilent 2100 bioanalyzer (Agilent Technologies, Santa Clara, CA, USA). The double-stranded PCR product was heat-denatured and circularized using a splint oligo. Single-strand circular DNA libraries were amplified via rolling circle replication with phi29 polymerase to produce DNA nanoballs. These were sequenced on a patterned nanoarray using combinatorial probe-anchor synthesis, generating single-end 50 (or paired-end 100/150) base reads. Triplicate samples were sequenced.

Genes were considered significantly up- or down-regulated if they met a univariate *p*-value of <0.05 and had an absolute fold difference of ≥1.5. A univariate significance threshold of 0.001 was used for filtering or pre-screening genes prior to comparison. Signaling pathway analysis was performed using DAVID (Version 6.7).

Significant KEGG pathways (adjusted *p*-value < 0.05) were visualized as dot plots using the clusterProfiler and ggplot2 R packages (Version R.4.5.1). The top up- or down-regulated categories were identified based on their statistical significance, and the number of genes contributing to each category was illustrated.

### 4.10. In Vivo Tumorigenesis Assays

Female athymic nude mice (4–6 weeks old) were purchased from the Jackson Laboratory. All animal procedures were conducted in accordance with the National Institutes of Health guidelines for the care and use of laboratory animals and were approved by the Institutional Animal Care and Use Committee (IACUC) at Case Western Reserve University. Mice were monitored daily for signs of pain and distress, including significant weight loss, decreased activity, and hunched posture. All animals were provided ad libitum access to food and water throughout the study.

MV4-11 cells (parental vs. resistant; mock vs. CHRNB4 overexpression; 3 × 10^6^) were suspended in a 3:7 mixture of serum-free medium and Matrigel^®^ (Corning, Inc.) and subcutaneously injected into both flanks of 6-week-old female athymic nude mice. Tumor growth was monitored every 2–3 days using an electronic caliper. Tumor volume was calculated using formula 1/2 × A × B × B, where A is the length and B is the width, expressed in cubic millimeters. Tumor growth curves were generated for each treatment group by plotting the mean tumor volume over time and AUC was calculated. The study endpoint was determined per IACUC guidelines.

Upon reaching the study endpoint, mice were euthanized by CO_2_ inhalation followed by cervical dislocation. Excised tumors were divided for different analyses. A portion was snap-frozen and stored at −80 °C for subsequent molecular characterization. Another portion was fixed in 10% neutral buffered formalin for 24 h at 4 °C for histological analysis. After fixation, tissues were transferred to PBS, processed, and embedded in paraffin blocks for immunohistochemical and hematoxylin and eosin staining.

### 4.11. Hematoxylin and Eosin (H&E) and Immunohistochemical (IHC) Staining

Tumor and tissue samples were harvested from mice and immediately preserved by fixation in 10% neutral buffered formalin. Following fixation, the samples were submitted to the Department of Pathology at the MetroHealth System, Case Western Reserve University, for processing and IHC (Ki-67) or H&E staining. The prepared slides were visualized and photographed with a Leica microscope (Leica Microsystems, Wetzlar, Germany) fitted with a high-resolution spot camera, which was interfaced with Image-Pro Plus software (Media Cybernetics, Rockville, MD, USA) for image acquisition.

### 4.12. Gene Expression and Survival Analysis

mRNA expression data for the CHRNB4 gene were obtained from The Cancer Genome Atlas (TCGA) AML cohort (TCGA, Firehose Legacy) via the cBioPortal for Cancer Genomics (https://www.cbioportal.org/ (accessed on 3 October 2025)). Patients were stratified into two groups based on their CHRNB4 mRNA expression levels: a low-expression group (lower 25% of expression values, i.e., the first quartile) and a high-expression group (remaining 75%). Overall survival (OS) was the primary endpoint. Survival curves were estimated using the Kaplan–Meier method, and differences between the groups were assessed using the log-rank test.

Survival analyses were conducted using the median expression value, optimal cutoff values, and a predefined 25% cutoff to stratify patients into expression groups. A multivariate Cox proportional hazards regression analysis was then performed to evaluate the prognostic impact of CHRNB4 expression alongside other relevant clinical variables. All statistical analyses were performed using JMP^®^ Student Edition version 18 (SAS Institute Inc., Cary, NC, USA).

### 4.13. Statistical Analysis

Statistical analyses were performed using JMP Student Edition (Version 18). Sample sizes were not statistically predetermined to ensure a specific power but were chosen based on the established literature for functional oncology assays. While randomization and blinding were not formally applied to experimental procedures, functional assays—including spheroid and colony frequency and dimensions—were quantified using Image-J/FIJI (Version 2.14.0; 1.54f) with a 50 µm diameter threshold and validated by an independent investigator blinded to experimental conditions. In vitro experiments, such as qPCR, Western blotting, and proliferation assays, were performed in biological triplicate (*n* = 3) unless otherwise noted.

Data were evaluated for normality via Shapiro–Wilk tests and for homogeneity of variance to justify parametric analyses. Group means were compared using two-tailed Student’s *t*-tests, with non-parametric alternatives applied for unequal variances; one-way ANOVA or mixed-effects models were utilized to account for intra-subject clustering in bilateral flank studies. In vivo growth kinetics were evaluated using Area Under the Curve (AUC) to emphasize cumulative tumor burden. Transcriptomic data were aligned with STAR, normalized by DESeq2, and screened using a Benjamini–Hochberg adjusted *p*-value (*p* < 0.05) followed by a post hoc fold-change threshold (|log2FC| > 1). Clinical relevance was assessed via multivariable Cox regression models adjusted for age, sex, and ELN 2022 risk categories using complete-case analysis. Finally, the association between CHRNB4 and venetoclax resistance was examined across primitive and monocytic maturation states to account for differentiation-driven sensitivity. In all cases, *p* < 0.05 was considered statistically significant.

## 5. Conclusions

In conclusion, our study identifies a potential BCL-2-independent mechanism associated with venetoclax resistance in AML, suggesting a shift in focus from classical apoptotic blockades toward the NLRI pathway. We demonstrate that the downregulation of CHRNB4 correlates with aggressive tumor phenotypes and heightened tumorigenicity in our experimental models. By establishing that enforced CHRNB4 expression can revert these malignant traits and impair tumor growth in vivo, we provide exploratory evidence of its functional significance as a therapeutic vulnerability. While these findings are currently model-specific and require further validation using standard pharmacological resistance metrics, the correlation between CHRNB4 suppression and poor clinical outcomes suggests its potential as a predictive biomarker for patient stratification. Collectively, these findings provide a preliminary rationale for investigating the NLRI axis to address drug resistance and offer a framework for developing next-generation combination therapies to improve the prognosis of patients with refractory AML.

## Data Availability

All relevant data supporting the key findings of this study were available from the corresponding authors upon reasonable request. Partial data were obtained from “Public data analysis” including TCGA.

## Supplementary Materials

The following supporting information can be downloaded at https://www.mdpi.com/article/10.3390/cancers18081187/s1, Figure S1: BCL-2 pathway independence in VEN-resistance development. (A,C) Densitometric quantification of representative Western blot bands from Figure 1G,H. Results are expressed as mean ± SD. (B,D) Full, uncropped Western blot images corresponding to the data shown in Figure 1G,H. Note: Membranes were cut into strips prior to incubation to resolve targets with similar molecular weights, overlapping non-specific bands, or shared secondary antibody species. Figure S2: VEN-resistant cells have higher tumorigenic potential in vivo. (A) Graphs are the densitometric quantification of Western blot bands from Figure 2H. Data are presented as mean ± SD. (B) Full, uncropped Western blot images corresponding to the data shown in Figure 2H. Note: Due to overlapping signal or shared host species, membranes were cut into strips to ensure target specificity. Figure S3: Expression of CHRNB4 in VEN-resistant or sensitive cells. (A,C) Graphs are the densitometric quantification of Western blot bands from Figure 4C,E. Data are presented as mean ± SD. (B,D) Full, uncropped Western blot images corresponding to the data shown in Figure 4C,E. Note: To avoid signal interference from non-specific bands or shared secondary antibodies, membranes were cut into strips for independent target detection. Figure S4: Expression of CHRNB4 in AML cells transfected with CHRNB4 expression vector. (A,C) Graphs are the densitometric quantification of Western blot bands from Figure 5C,E. Data are presented as mean ± SD. (B,D) Full, uncropped Western blot images corresponding to the data shown in Figure 5C,E. Note: For targets with similar molecular weights or overlapping bands, membranes were cut into strips prior to antibody incubation to maintain signal clarity. Table S1: Differential expressed transcripts in VEN-resistant vs. parental Kasumi-1 cells. Table S2: Differential expressed transcripts in VEN-resistant vs. parental MV4-11 cells. Table S3: Top 100 differential expressed transcripts in VEN-resistant vs. parental Kasumi-1 cells. Table S4: Top 100 differential expressed transcripts in VEN-resistant vs. parental MV4-11 cells. Table S5: Lists of KEGG pathways in VEN-resistant vs. parental Kasumi-1 and MV4-11 cells. Table S6: Lists of genes within NLRI pathways in Kasumi-1 and MV4-11 cells. Table S7: AML patient characteristics and CHRNB4 expression levels (TCGA cohort).
