# Supplementary material for: CHRNB4-Mediated Neuroactive Signaling Rewiring Drives Adaptive Resistance to BCL-2 Inhibition in Acute Myeloid Leukemia
Source: Cancers (Basel). 2026 Apr 8;18(8):1187. doi: 10.3390/cancers18081187 (PMC13115082; doi:10.3390/cancers18081187)
Supplement: Supplementary file 1 [file cancers-18-01187-s001.zip › cancers-4211798-Figure S1-S4.pdf]

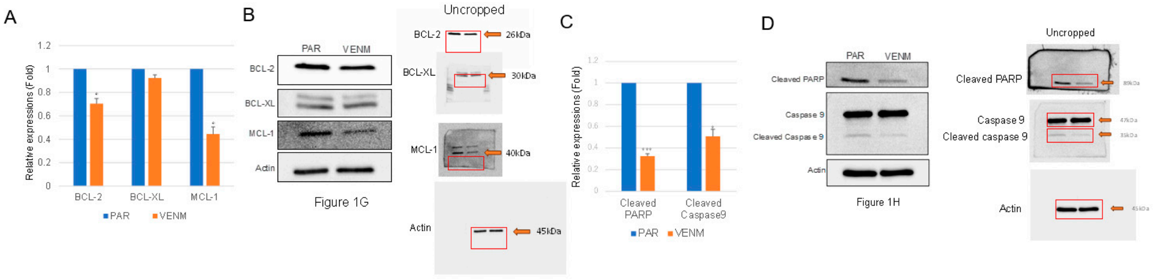

Figure S1. BCL-2 pathway independence in VEN-resistance development. (A,C) Densitometric quantification of representative Western blot bands from Figure 1G,H. Results are expressed as mean ± SD. (B,D) Full, uncropped Western blot images corresponding to the data shown in Figures 1G,H. Note: Membranes were cut into strips prior to incubation to resolve targets with similar molecular weights, overlapping non-specific bands, or shared secondary antibody species.

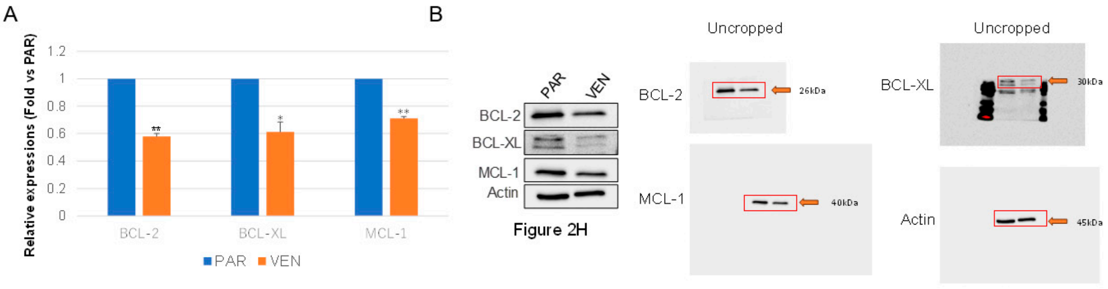

Figure S2. VEN-resistant cells have higher tumorigenic potential in vivo. (A) Graphs are the densitometric quantification of Western blot bands from Figure 2H. Data are presented as mean ± SD. (B) Full, uncropped Western blot images corresponding to the data shown in Figure 2H. Note: Due to overlapping signal or shared host species, membranes were cut into strips to ensure target specificity.

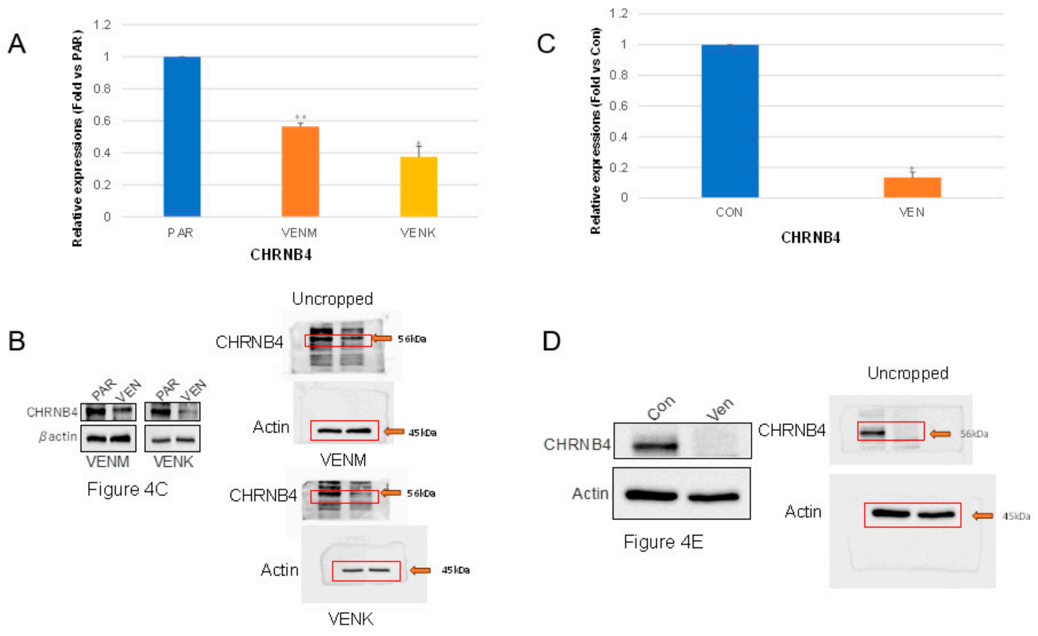

Figure S3. Expression of CHRNB4 in VEN-resistant or sensitive cells. (A,C) Graphs are the densitometric quantification of Western blot bands from Figure 4C,E. Data are presented as mean ± SD. (B,D) Full, uncropped Western blot images corresponding to the data shown in Figure 4C,E. Note: To avoid signal interference from non-specific bands or shared secondary antibodies, membranes were cut into strips for independent target detection.

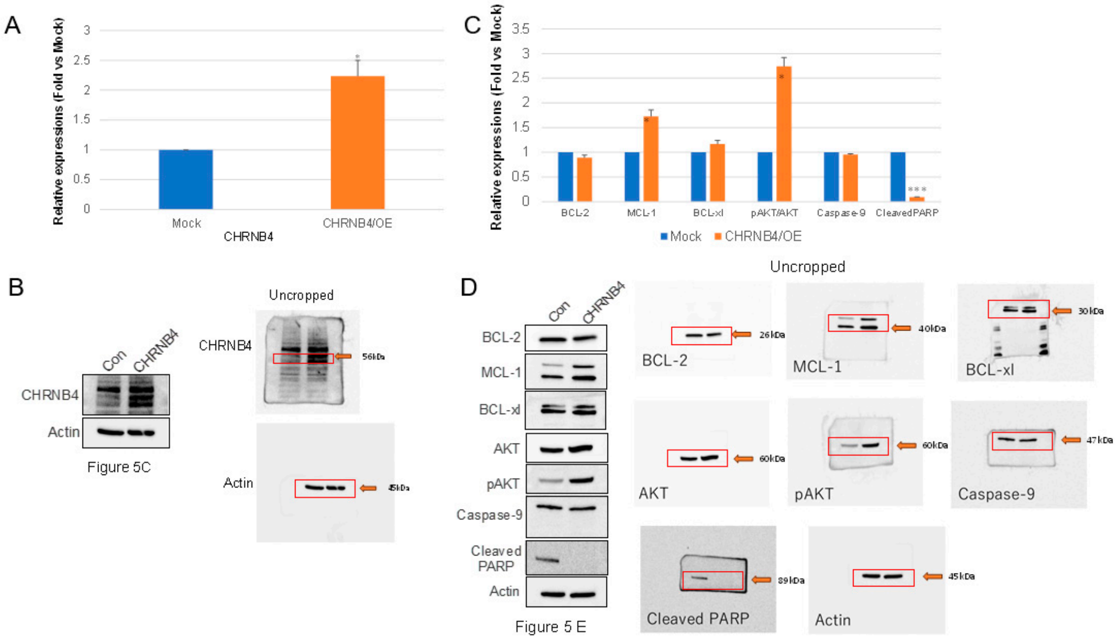

Figure S4. Expression of CHRNB4 in AML cells transfected with CHRNB4 expression vector. (A,C) Graphs are the densitometric quantification of Western blot bands from Figure 5C,E. Data are presented as mean ± SD. (B,D) Full, uncropped Western blot images corresponding to the data shown in Figure 5C,E. Note: For targets with similar molecular weights or overlapping bands, membranes were cut into strips prior to antibody incubation to maintain signal clarity.
