# Supplementary material for: CHRNB4-Mediated Neuroactive Signaling Rewiring Drives Adaptive Resistance to BCL-2 Inhibition in Acute Myeloid Leukemia
Source: Cancers (Basel). 2026 Apr 8;18(8):1187. doi: 10.3390/cancers18081187 (PMC13115082; doi:10.3390/cancers18081187)
Supplement: Supplementary file 1 [file cancers-18-01187-s001.zip › Suppl Table S7.pdf]

Supplementary Table S7: AML patient characteristics and CHRNB4 expression levels (TCGA cohort).

Characteristic	CHRNB4 expression		
	**Low**	**High**	**P**
	**n=43**	**n=130**	
**Age, years**			0.5386
Median	60	57.5	
Range	18-81	21-88	
**Sex, n (%)**			1
Male	23 (53.5)	70 (53.8)	
Female	20 (46.5)	60 (46.2)	
**Race, n (%)**			0.4343
White	37 (86.1)	119 (91.5)	
Black	5 (11.6)	8 (6.2)	
Asian	1 (2.3)	1 (0.8)	
Missing	0 (0)	2 (1.5)	
**CHRNB4 value**			<0.0001
Median	0	0.056653	
Range	0-0.008343	0.010064-1.324407	
**Status, n (%)**			0.5823
Dead	30 (69.8)	84 (64.6)	
Alive	13 (30.2)	46 (35.4)	
**Overall survival, months**			0.0043
Median	7.01	12.02	
Range	0-54.96	0-93.99	
